# Comparative analysis of the effectiveness of coarctation surgery between neonates and infants

**DOI:** 10.1590/1806-9282.20231626

**Published:** 2024-05-20

**Authors:** Mustafa Yilmaz, Başak Soran Turkcan, Ata Niyazi Ecevit, Yasemin Özdemir Şahan, Atakan Atalay

**Affiliations:** 1 Ankara Bilkent City Hospital, Department of Pediatric Cardiovascular Surgery – Ankara, Turkey.; 2 Ankara Bilkent City Hospital, Department of Pediatric Cardiology – Ankara, Turkey.

**Keywords:** Coarctation, Newborn, Infant

## Abstract

**OBJECTIVE:**

This study aimed to compare the effectiveness of resection and extended end-to-end anastomosis between neonate and infant patients with coarctation.

**METHODS:**

This study was designed retrospectively and included 41 neonate (<30 days) and infant (30 days to 1 year) patients who were operated on using the resection and extended end-to-end anastomosis technique for aortic coarctation. Preoperative aortic annulus diameters and Z scores, all aortic arch diameters and Z scores, the presence of hypoplastic aortic segment, and the presence of prematurity were reviewed in both groups. Subsequently, we investigated whether these parameters were statistically related to the residual gradient in the operation area, whether there was a need for early re-intervention, and what was the incidence of mortality in the early postoperative period. In addition, the aortic arch Z scores of the patients at 6 months postoperatively were examined.

**RESULTS:**

While the mean age (p<0.001), body weight (p<0.001), and proximal arch Z score (p=0.029) were found to be significantly lower in the neonate group than in the infant group, the total length of the intensive care unit stay (p=0.013) and the total length of hospital stay (p=0.017) were found to be significantly higher. In addition, significant enlargement was detected in the proximal arch, distal arch, and isthmus segments in both patient groups.

**CONCLUSION:**

The resection and extended end-to-end anastomosis is an equally effective technique that can provide a marked decrease in gradient in the coarctation area and a significant enlargement of the aortic arch segments in the early period after coarctation repair in both neonate and infant patients.

## INTRODUCTION

Aortic coarctation is a congenital anomaly characterized by discrete or diffuse stenosis in the juxtaductal region of the descending aorta. It constitutes approximately 10% of all cases of congenital heart diseases^1^.

The resection and extended end-to-end anastomosis (REEA) technique is a very useful procedure that provides resection of the coarctated region and expands the hypoplastic distal arch and isthmus. However, as the aorta of neonate patients with hypoplastic arch is very small in diameter and prone to rupture, effective utilization of this technique requires extensive experience. Therefore, to minimize the difficulty of the operation, some clinics postpone surgeries for hemodynamically stable neonates until the infancy period to allow the cardiac structures to grow.

Our aim in this study was to compare the coarctation patients who had undergone coarctation surgery using the REEA technique, in the neonatal and infant period, in terms of early surgical results in the postoperative period. These included the need for re-intervention, development of early recoarctation, growth of the aortic arch segments, and in-hospital mortality. Thus, it was investigated whether the timing of the operation had an effect on the postoperative results.

## METHODS

This was a retrospective observational study that included pediatric patients who were operated on with a left posterolateral thoracotomy using the REEA technique, between March 2019 and December 2022, in Ankara Bilkent City Hospital Pediatric Cardiovascular Surgery department. The study was approved by the institutional ethics committee of the same institution with approval number E2-23-3744. The patients were classified into two groups: neonates (<30 days) and infants (30 days to 12 months). All patients were further examined to determine whether they had isolated coarctation or coarctation accompanied by hypoplastic aortic arch. An exclusion criterion in the study was the presence of additional major cardiac anomaly, which was hemodynamically significant and required additional surgical intervention.

Preoperative demographic and echocardiographic data of the patients were collected. For this purpose, the data reporting the patients’ body weight, age, presence of prematurity, presence of genetic syndrome, diameter, and Z score of aortic annuli as well as Z scores of proximal arch, distal arch, and isthmus were all gathered. Cardiovascular structures with a Z score below −2 were defined as hypoplastic.

In accordance with the common consensus of cardiology and cardiovascular surgery clinics, endovascular balloon dilatation was applied to the patients who required urgent intervention in the neonatal period due to hemodynamic instability. Other patients diagnosed in our clinic or referred to our institution were scheduled for surgery as soon as possible without any intentional delay.

Both arm-leg systolic blood pressure gradient (ALSG) and maximum systolic echocardiographic gradients in the repaired coarctation and/or hypoplastic aortic arch region of the patients were recorded within the first week after surgery. A simultaneous systolic gradient above 20 mm Hg in both of them was considered diagnostic in terms of recurrent coarctation. These measurements were repeated at the sixth-month follow-up visit of the patients following their discharge. In addition, aortic annulus, proximal arch, distal arch, and isthmus Z scores were also calculated and compared with preoperative values.

### Statistical analysis

Mean and standard deviation were calculated in descriptive statistics of continuous variables. In the definition of categorical variables, frequency (n) and percentage (%) values were used. Normality assumptions of the variables were examined with the Shapiro-Wilk test. The Mann-Whitney U test was used to compare continuous variables between the two groups, and the chi-square/Fisher's exact analysis was used to determine the relationships between categorical variables. The IBM SPSS.25 program was used in all analyses, and the p<0.05 value was accepted as the significance level.

## RESULTS

A total of 41 patients, including 22 (53.7%) neonates and 19 (46.3%) infants, were included in the study. A comparison of demographic and clinical data of neonate and infant patients is shown in Table 1.

**Table 1:** Demographic and clinical data.

Parameters		Neonate (<30 days)	Infant (>30 days)	p
n (%)		22 (53.7)	19 (46.3)	
Age (days)*		20.86±9.69	112.89±47.53	**<0.001**
Weight (kg)*		3.33±0.57	5.32±1.28	**<0.001**
Gender n (%)**				0.436
	Male	19 (86.4)	14 (73.7)	
	Female	3 (13.6)	5 (26.3)	
Genetic anomaly (%)				0.321
	Yes	21 (95.5)	16 (84.2)	
	No	1 (4.5)	3 (15.8)	
Prematurity n (%)***				0.524
	No	17 (77.3)	13 (68.4)	
	Yes	5 (22.7)	6 (31.6)	
Preoperative cardiac Z scores				
	Aortic annulus Z score*	-1.99±1.50	-1.22±2.12	0.284
	Proximal arc Z score*	-3.94±1.49	-2.99±1.42	**0.029**
	Distal arc Z score*	-3.91±1.73	-3.43±1.51	0.472
	Istmus Z score*	-4.68±2.52	-3.39±1.52	0.094
Hypoplastic arcus n (%)**				0.080
	No	1 (4.5)	5 (26.3)	
	Yes	21 (95.5)	14 (73.7)	
Length of stay at intensive care unit (days)*		2.55±0.69	2.14±0.97	**0.013**
Total length of hospitalization (days)*		14.23±5.03	11.24±6.34	**0.017**
Mortality n (%)*				0.610
	No	19 (86.4)	18 (94.7)	
	Yes	3 (13.6)	1 (5.3)	
Postoperative re-intervention requirement n (%)*				0.610
	No	19 (86.4)	18 (94.7)	
	Yes	3 (13.6)	1 (5.3)	

* Mann-Whitney U test;

** Fisher's exact test;

*** Chi-square test.

The mean age of the neonate group (20.86±9.69 days vs 112.89±47.53 days) (p<0.001), body weight (3.33±0.57 kg vs 5.32±1.28 kg) (p<0.001), and proximal arch Z score (−3.94±1.49 vs −2.99±1.42) (p=0.029) were significantly lower than the infant group, while the duration of intensive care unit stay (2.55±0.69 days vs 2.14±0.97 days) (p=0.013) and total hospitalization time (14.23±5.03 days vs 11.24±6.34 days) (p=0.017) was found to be significantly higher. On the contrary, no significant relationship was found between the neonate and infant groups in terms of the presence of genetic anomalies and prematurity (p=0.32 and p=0.52).

While hypoplasia (Z score<-2.0) was detected in at least one segment of the aortic arch in 95.5% of neonate patients, this rate was 73.3% in infant patients (p=0.08).

In the postoperative period, three neonates (13.6%) and one infant (5.3%) died. The underlying cause of death in all patients was multiorgan failure secondary to sepsis. No significant stenosis was observed in the operative area in the postoperative echocardiographic examinations of these patients.

An interventional procedure was performed in three patients (13.6%) in the neonate group and in one patient (5.3%) in the infant group due to the development of recoarctation within the first 6 months postoperatively.

As shown in Table 2 in both neonate and infant groups, patients with an ALSG and preoperative systolic max ECHO gradient above 20 mm Hg in the coarctation area had significant decreases in the postoperative measurements. However, it was observed that these values persisted above 20 mm Hg in two of the neonates and in three of the infant patients (p<0.001). In two of these patients, surgical reintervention was immediately performed after the first coarctation repair due to the presence of high proximal blood pressure, insufficient distal perfusion, and hemodynamic instability in the intensive care unit. Both of these reoperated patients were neonates and had a high systolic max ECHO gradient (>70 mm Hg) at the anastomosis region, accompanied by significant diastolic extension in the descending aorta and diminished lower extremity pulses in the postoperative evaluation. In the reoperation procedure, an aortoplasty using a bovine pericardial patch was performed on the previous operative area. The systolic gradients of these patients regressed below 20 mm Hg right after the second operation, and their follow-up was uneventful. The remaining one neonate and three infant patients whose ALSG and maximum systolic anastomosis gradients were above 20 mm Hg were not intervened due to a lack of distal perfusion disturbances and any diastolic extension in the descending aorta. In two of these infant patients, the gradients rapidly decreased below 20 mm Hg in the following postoperative days. On the contrary, in one neonate and one infant patient, signs of left ventricular hypertrophy developed, and high anastomosis gradients with diastolic extension in the descending aorta persisted. In line with the institutional first-line treatment strategy in recoarctation, balloon angioplasty was performed in the third and fourth months after the operation, respectively. In these patients, ALSG and the maximum systolic anastomosis gradient was successfully declined below 20 mm Hg in the recoarctation area and the diastolic tail in the descending aorta disappeared. In both groups, once the pre- and early ALSG and postoperative maximum systolic ECHO gradients were compared, significant gradient decrease was detected (p<0.001 vs p<0.001). On the contrary, no significant difference was found between the ALSG and postoperative early and sixth month systolic max ECHO gradients for both groups (p=1.0 vs p=1.0).

**Table 2:** Comparison of arm-leg blood pressure systolic gradient and systolic max echo gradients obtained at different times in neonates and infants.

Neonate		ALSG and postoperative early systolic max echo gradient (mm Hg)*		p
ALSG and preoperative Sistolik max echo gradient (mm Hg)*		<20 mm Hg (n, %)	≥20 mm Hg (n, %)	**<0.001**
	< 20 mm Hg (n, %)	1 (5.0)	0 (0.0)	
	≥ 20 mm Hg (n, %)	19 (95.0)	2 (100.0)	
		ALSG and postoperative sixth-month systolic max echo gradient (mm Hg)*		1.00
ALSG and postoperative early systolic max echo gradient (mm Hg)*		<20 mm Hg (n, %)	≥20 mm Hg (n, %)	
	<20 mm Hg (n, %)	19 (90.5)	1 (100.0)	
	≥20 mm Hg (n, %)	2 (9.5)	0 (0.0)	
Infant		ALSG and postoperative early systolic max echo gradient (mm Hg)*		p
ALSG and preoperative systolic max echo gradient (mm Hg)*		<20 mm Hg (n, %)	≥20 mm Hg (n, %)	**<0.001**
	<20 mm Hg (n, %)	1 (6.3)	0 (0.0)	
	≥20 mm Hg (n, %)	15 (93.8)	3 (100.0)	
		ALSG and postoperative sixth-month systolic max echo gradient (mm Hg)*		
ALSG and postoperative early systolic max echo gradient (mm Hg)*		<20 mm Hg (n, %)	≥20 mm Hg (n, %)	1.00
	<20 mm Hg (n, %)	15 (88.2)	1 (50.0)	
	≥20 mm Hg (n, %)	2 (11.8)	1 (50.0)	

* Fisher's exact test. ALSG: arm-leg blood pressure systolic gradient.

As shown in Table 3, Z scores of the aortic annulus (p=0.002), proximal arch (p<0.001), distal arch (p=0.001), and isthmus (p=0.001) in the neonate group were observed to be significantly increased when compared with those in the sixth month postoperatively. Similarly, Z scores of the same structures in the infant group have also significantly increased (p<0.001). However, no significant change was detected in the infant group in terms of annulus Z score between preoperative and postoperative 6-month periods (p>0.05).

**Table 3:** Comparison of preoperative and postoperative sixth-month Z score parameters in neonatal and ınfant groups.

		Preoperative Z score	Postoperative sixth-month Z score	p
Neonate				
	Aortic annulus	-1.99±1.50	-1.00±0.93	**0.002**
	Proximal arc	-3.94±1.49	-1.27±1.07	**<0.001**
	Distal arc	-3.91±1.73	-1.63±1.61	**0.001**
	Istmus	-4.68±2.52	-2.08±1.96	**0.001**
Infant				
	Aortic annulus	-1.22±2.12	-0.26±1.10	0.094
	Proximal arc	-2.99±1.42	-1.04±1.09	**<0.001**
	Distal arc	-3.43±1.51	-0.53±1.21	**<0.001**
	Istmus	-3.39±1.52	-1.19±0.47	**<0.001**

Statistically significant p-value are indicated in bold.

## DISCUSSION

According to the contemporary literature, the REEA technique through left posterolateral thoracotomy is accepted as one of the most preferred surgical approaches in coarctation, whether isolated or accompanied by a hypoplastic arch. However, there is an ongoing debate about the surgical method to be applied in cases where aortic coarctation is accompanied by hypoplasia of the proximal arch of the aorta. Many researchers state that if the hypoplastic proximal arch is not intervened on in the first surgical operation, recoarctation may develop in the medium and long term, re-intervention may be required, and additional morbidities, such as persistent hypertension, may develop. Mery et al., performed arch reconstruction and coarctation repair using the “aortic arch advancement” technique with median sternotomy, CPB, and antegrade selective perfusion support, on 275 neonate and infant patients under the age of 1 year with a proximal arch Z score average of −5.06. Perioperatively, eight patients (3%) died. The surviving patients were followed for an average of 6 years, and the need for re-intervention was observed in eight of these patients (3%)^2^. Other researchers have published similar results^3^. On the contrary, some researchers argue that unless the proximal arch Z score is below −4.5, additional intervention is unnecessary and the proximal arch will develop progressively with the high blood flow provided after the removal of the coarct segment with the REEA technique and the enlargement of the distal arch and isthmus. In this study, we found that in cases where the proximal arch was hypoplasic and only the distal arch and isthmus were enlarged by applying the REEA technique, in the postoperative sixth-month imaging, there was no significant gradient in the operated area, the Z scores of all aortic arch segments developed significantly, and the need for re-intervention was not observed.

In the literature, many risk factors that may cause reintervention after coarctation repair have been reported. These can be listed as operating age, operating weight, presence of prematurity, underdevelopment of left heart structures, association of hypoplastic arch, and surgical technique applied^4–6^. It has been shown that surgical repair performed in the neonatal period has a positive effect on the development of left heart structures such as the left ventricular outflow tract, mitral and aortic valve annulus dimensions, and aortic arch in the long term and gives more favorable results than repairs performed in the infant period, in terms of preventing persistent hypertension^1^. Therefore, in many clinics around the world, coarctation repair is performed as soon as possible after diagnosis. However, some researchers suggest that surgical repair of coarctation in neonates should be postponed to infancy because of its own disadvantages. These can be listed as follows: coarctation repair in neonates is technically more difficult than infant repair, as the aortic arch and descending aorta are much smaller in size than infants and are more prone to rupture. In neonates and, in particular, in low-birth-weight patients, it is very critical to perform the anastomosis once and flawlessly in order to avoid complications such as acute bleeding after anastomosis or recoarctation in the chronic period. In addition, the ductus arteriosus is more fragile in neonates and complete resection of ductal tissue remnants from the entire aortic segment is more challenging^7^. However, despite all these difficulties, the early and long-term results of aortic coarctation repairs performed in the neonatal period are very successful because of improved surgical techniques and surgical instruments. In this study, the operative age and operative weight of the neonate patients were found to be significantly lower than the infants. However, it was found that the gradient in the coarctation region decreased significantly in the early period after surgical repair in both groups, and this situation persisted significantly at the postoperative sixth month. In both groups, it was observed that in addition to coarctation in the aortic arch, the hypoplastic arch also accompanied prominently, and there was no significant difference between the two groups in this regard. Moreover, the need for re-intervention after the surgery was similarly low in both age groups.

According to a study conducted with the data of coarctation patients treated in 52 hospitals in the United States, the presence of prematurity and low birth weight may lead to both prolonged hospitalization and increased in-hospital mortality. In addition, each 30-day delay in hospitalization of the patient reduces the postoperative hospital stay of the patient by 2.6 days^8^. IJsselhof et al., compared neonate and infant patients who underwent coarctation repair and found that neonate patients have required significantly longer intensive care and total hospitalization, compared with infants. It has been stated that the reason for this situation may be the more severe form of coarctation seen in neonate patients, and therefore their recovery period may be longer^1^. In our opinion, this situation is due to the prematurity of the heart and lung structures of the neonates compared with the infant patients, and accordingly, they require non-invasive respiratory support longer in the intensive care unit after extubation. Despite the lack of statistical significance, in our study, a higher mortality was observed in neonates compared with infant patients, and these data were similar to other series in the literature.

## CONCLUSION

The REEA technique provides equally successful results in neonate and infant patients with coarctation, with a low rate of re-intervention and significant reduction of postoperative systolic maximum gradient at the coarctation area. It was observed that the hypoplastic aortic arch segments repaired using this technique enlarged rapidly in the early postoperative period in both groups. Although the total length of intensive care unit stay and total length of hospital stay were significantly longer in neonate patients than in infants, the REEA technique enables coarctation repair with low mortality in both age groups^9^.
